# Betulinic Acid Ameliorates T-2 Toxin-Induced Neuroinflammation by Suppressing Oxidative Stress via Regulating Nrf2/NLRP3 Axis

**DOI:** 10.3390/vetsci13060509

**Published:** 2026-05-24

**Authors:** Jiao Wu, Hongyi Ding, Jiayu He, Zhaoping Ou, Ziming Wang, Wenyu Ba, Quanwei Li, Yunqiu Yan, Jiaming Wang, Jine Yi, You Huang

**Affiliations:** 1 Hunan Engineering Research Center of Livestock and Poultry Health Care, College of Veterinary Medicine, Hunan Agricultural University, Changsha 410128, China; wujiao@stu.hunau.edu.cn (J.W.); dinghongyi@stu.hunau.edu.cn (H.D.); hejiayu@stu.hunau.edu.cn (J.H.); ouzhaoping@stu.hunau.edu.cn (Z.O.); 1602565506@stu.hunau.edu.cn (Z.W.); 1545055405@stu.hunau.edu.cn (W.B.); liqw@hunau.edu.cn (Q.L.); 13975881386@126.com (Y.Y.); 2 Hunan Xinwufeng Co., Ltd., Changsha 410005, China; hanjingfei1988@126.com; 3 School of Life Sciences and Medical Technologies, Hainan Medical University, Hainan Academy of Medical Sciences, Haikou 571199, China

**Keywords:** T-2 toxin, Betulinic acid, neuroinflammation, Nrf2/NLRP3 signaling pathway

## Abstract

T-2 toxin is widely present in agricultural products and poses a significant neurotoxicity threat. Betulinic acid (BA), a natural triterpenoid, exhibits strong antioxidant and anti-inflammatory properties. However, its protective role against T-2 toxin-induced neuroinflammation remains poorly understood. This study aimed to elucidate the mechanisms underlying T-2 toxin-induced neurotoxicity and evaluate the therapeutic potential of BA. Our results demonstrated that T-2 toxin (1 mg/kg/bw) exposure caused significant pathological damage in the hippocampus and cerebral cortex. T-2 toxin also induced marked oxidative stress, reflected by elevated reactive oxygen species (ROS) accumulation. At the inflammatory level, T-2 toxin upregulated the mRNA expression of pro-inflammatory cytokines (Interleukin-1 beta (IL-1β), Interleukin-6 (IL-6)) and altered anti-inflammatory IL-10 expression. In addition, T-2 toxin exhibited strong binding affinity for the tight junction proteins Occludin and Claudin-1 (docking energies of −4.41 and −5.53 kcal/mol, respectively), and molecular dynamics simulations confirmed stable protein–ligand interactions. At the molecular level, T-2 toxin suppressed Nuclear factor erythroid 2-related factor 2 (Nrf2) protein expression, increased Kelch-like ECH-associated protein 1 (Keap1) expression, and activated the NOD-like receptor family pyrin domain containing 3 (NLRP3) inflammasome pathway. Furthermore, molecular docking analysis revealed that BA displayed strong binding affinity to proteins associated with the blood–brain barrier and the Nrf2/NLRP3 signaling pathway. Collectively, these findings indicate that BA mitigates T-2 toxin-induced neuroinflammation through regulating the Nrf2/NLRP3 signaling pathway in mice. Not only do these results clarify a key mechanism of T-2 toxin-induced central nervous system injury, but they also highlight BA as a promising candidate for developing interventions targeting mycotoxin-related neurological disorders.

## Simple Summary

T-2 toxin is widely found in agricultural products. Although the neurotoxicity of T-2 has been reported, the key molecules and underlying regulatory mechanisms remain unclear. Betulinic acid (BA), a natural triterpenoid from birch bark, possesses strong antioxidant and anti-inflammatory properties. The mechanisms of BA and T-2 toxin in neuroinflammation remain elusive. Therefore, we established a T-2 toxin-exposed mouse model to explore the protective role of BA against T-2 toxin-induced brain damage. We demonstrated that BA could alleviate T-2 toxin-induced hippocampal and cortical injury, oxidative stress, and an imbalance in inflammatory cytokines by restoring BBB integrity, reducing ROS, and regulating the Nrf2/Keap1 signaling pathway. Collectively, these findings confirmed that BA mitigated T-2 toxin-induced neurotoxicity through regulation of the Nrf2/NLRP3 axis, providing new insights into the mechanisms of T-2-induced central nervous system (CNS) injury and potential therapeutic ways.

## 1. Introduction

T-2 toxin, a prevalent foodborne mycotoxin produced by *Fusarium sporotrichioides*, is a frequent contaminant of barley, maize, wheat, and maize-based animal feeds [1]. Due to its pronounced lipophilicity, T-2 toxin can be efficiently absorbed through dermal, gastrointestinal, and other exposure routes in humans and animals [2], thereby exerting broad-spectrum toxicological effects that pose significant risks to public and animal health. Recent research findings indicated that T-2 toxin could cross the blood–brain barrier and cause neurotoxicity in the brain [3]. Exposure to T-2 toxin has been shown to induce abnormally elevated markers of oxidative stress in the brain, accompanied by typical neurotoxic manifestations such as cognitive dysfunction [4,5]. Meanwhile, administration of T-2 toxin at a dose of 4 mg/kg/bw could induce neuronal atrophy, cell swelling, pericellular space enlargement, extensive hemorrhage, and apoptosis in the cerebral cortex of mice [6]. Given that exposure to T-2 toxin in the food and feed chain is difficult to avoid and cannot be fully controlled through processing-based detoxification strategies, increasing attention has been directed toward the use of bioactive natural compounds to mitigate T-2 toxin-induced toxicity.

Natural bioactive compounds, including polyphenols, flavonoids, terpenoids, alkaloids, and polysaccharides, exhibit strong antioxidant and anti-inflammatory properties that help counteract toxin-induced oxidative stress and inflammation [7]. Rosmarinic acid (RA), a phenolic compound commonly found in *Rosmarinus officinalis*, *Perilla frutescens,* and *Melissa officinalis*, has demonstrated protective effects against T-2 toxin–induced intestinal injury by reducing oxidative stress and inflammation [8]. In addition, selenomethionine effectively mitigated T-2 toxin-induced immunotoxicity by exerting strong antioxidant and anti-inflammatory effects [9]. Thus, plant extracts and plant-derived bioactive molecules could effectively reduce the organ toxicity of T-2 toxin. Betulinic acid (BA) is a naturally occurring pentacyclic triterpenoid mainly found in birch bark and other woody plants, which has a wide range of pharmacological properties, including antioxidant, anti-inflammatory, antitumor, antiviral, and immunomodulatory effects [10]. In the central nervous system, BA exhibited neuroprotective effects by reducing neuroinflammation, neuronal apoptosis, and memory impairment in models of neurodegenerative diseases. However, BA’s ability to ameliorate neuroinflammation induced by T-2 toxin exposure has not been confirmed.

Excessive oxidative stress is a fundamental mechanism driving neuronal injury during mycotoxin exposure. Mycotoxins such as T-2 toxin and ochratoxin A disrupt mitochondrial function, leading to excessive generation of reactive oxygen species (ROS), lipid peroxidation, protein oxidation, and DNA damage [11,12]. The sustained oxidative imbalance activates downstream inflammatory signaling, thereby creating a self-reinforcing loop between oxidative stress and neuroinflammation [13]. Nuclear factor erythroid 2–related factor 2 (Nrf2) acts as a central regulator of antioxidant defense, which is sequestered in the cytoplasm by Kelch-like ECH-associated protein 1 (Keap1). Under the condition of oxidative stress, Nrf2 dissociates from Keap1, translocates into the nucleus, and binds to the antioxidant response element (ARE) to activate genes such as heme oxygenase-1 (HO-1) and NAD(P)H quinone dehydrogenase 1 (NQO1). Activation of the Nrf2/HO-1 signaling pathway mitigated mycotoxin-induced oxidative injury by enhancing antioxidant capacity [14]. Extensive research has established that Nrf2 serves as a critical regulator of neuroinflammation by maintaining redox balance and suppressing the secretion of pro-inflammatory cytokines. Activation of Nrf2 inhibited microglial overactivation and reduced the release of cytokines such as interleukin-1 beta (IL-1β) and tumor necrosis factor-alpha (TNF-α) through downregulation of the nuclear factor kappa-B (NF-κB) and nucleotide-binding oligomerization domain-like receptor family pyrin domain-containing 3 (NLRP3) inflammasome pathways, thereby mitigating oxidative stress–induced neuronal injury [15]. In contrast, Nrf2 deficiency exacerbated neuroinflammatory responses and accelerated neurodegeneration in experimental models of Parkinson’s and Alzheimer’s diseases [16,17]. Meanwhile, BA could attenuate cyclophosphamide, Lipopolysaccharide, and zearalenone-induced inflammatory damage through Nrf2-dependent mechanisms [18,19,20]. It has been reported that BA reduced the secretion of IL-1β and IL-18 by inhibiting NLRP3 protein expression and the cleavage of caspase-1 [21]. However, whether BA could alleviate T-2 toxin-induced neuroinflammation by modulating Nrf2 and NLRP3 signaling pathways remains unclear.

In this study, we established a mouse model of T-2 toxin-induced neurotoxicity to mimic neuroinflammatory injury in the brain. We aimed to investigate the protective effects of BA against T-2 toxin-induced neuroinflammation and the underlying regulatory mechanisms, thereby providing potential therapeutic targets for the prevention and treatment of mycotoxin-associated neurological disorders.

## 2. Materials and Methods

### 2.1. Establishment of Animal Models

To explore the effect of T-2 toxin exposure on neuroinflammation, a total of thirty-two male C57BL/6J mice (7 weeks old, body weight: 17–20 g) were randomly assigned to four groups: control, low-dose (0.5 mg/kg/bw), medium-dose (1 mg/kg/bw), and high-dose (2 mg/kg/bw) T-2 toxin groups. Male mice were exclusively used in this experiment to avoid potential interference with experimental indicators caused by the estrous cycle and sex hormone fluctuations in female mice, reduce individual differences, and improve the reliability of the experimental data. A mixture of alcohol and PBS (1:12.5, *v*/*v*) was administered to the control group, whereas the other groups received T-2 toxin dissolved in the same vehicle for 14 consecutive days. On day 14, after an 8-hour fasting period, the mice were euthanized, and blood and brain samples were collected.

To explore the protective effect of BA against T-2 toxin-induced neuroinflammation in mice, thirty-five male C57BL/6J mice were randomly assigned into five groups (*n* = 7 per group): a CON group, a T-2 toxin group (1 mg/kg/bw), and three T-2 toxin + BA intervention groups receiving low (0.25 mg/kg/bw), medium (0.5 mg/kg/bw), or high (1 mg/kg/bw) doses of BA [22]. During the first week, mice in the CON and T-2 toxin groups were gavaged with an equivalent volume of soluble starch, while mice in the BA groups received their respective doses of BA. From day 8 to day 21, T-2 toxin (1 mg/kg/bw) was administered by gavage to the T-2 toxin group and all BA-treated groups, whereas the CON group received an equal volume of 1% soluble starch mixed with alcohol-PBS. BA was administered once daily by gavage for a total of 21 days. BA was purchased from Sigma (St Louis, MO, USA). T-2 toxin was obtained from Pribolab Biotechnology Co., Ltd. (Qingdao, China).

All animals were supplied by Hunan Sleck Jing da Laboratory Animal Co., Ltd., Changsha, China. and maintained under standard laboratory conditions (12-hour/12-hour light/dark cycle, 18–22 °C, 50–70% relative humidity). All reagents used in the experiments are listed in the Supplementary Materials (Supplementary Table S1). All animal procedures were approved by the Ethics and Use of Laboratory Animals Committee of Hunan Agricultural University and conducted in accordance with the Guidelines for Ethical Review of Laboratory Animal Welfare (Ethical approval number: 2023134).

### 2.2. Brain and Body Weight Measurement

The brain tissue was carefully and promptly separated, with the meninges and attached blood vessels removed. The brain weight was accurately measured using an electronic balance. The brain-to-body-weight ratio (brain weight/body weight × 100%).

### 2.3. Hematoxylin and Eosin (H&E) Staining

Brain tissue was fixed in 4% paraformaldehyde, then extracted and embedded in paraffin. Paraffin sections (5 μm thick) were cut, stained with H&E, and observed under a light microscope (Nikon Eclipse Ci, Tokyo, Japan).

### 2.4. Transmission Electron Microscopy (TEM)

Fresh brain tissue was fixed in 2.5% glutaraldehyde solution for 3 hours, followed by dehydration, embedding, and sectioning. Ultrathin sections (60–80 nm) were stained overnight at room temperature with uranyl acetate and lead citrate. The images were then analyzed and observed under a transmission electron microscope (Philips CM-100, Eindhoven, The Netherlands).

### 2.5. ROS Detection

Based on our previous study, the ROS level in the brain was measured using the oxidative fluorescent dye dihydroethidium (DHE) [22]. The brain tissue was rapidly collected and flash-frozen in liquid nitrogen, then sectioned using a cryostat. ROS production in the frozen sections was detected using DHE. Red fluorescence from the oxidized form of DHE was observed under a fluorescence microscope at an excitation wavelength of 535 nm and an emission wavelength of 610 nm. Images were captured using Image-Pro Plus 6.0 software, and quantitative analysis was performed using the software’s built-in analysis tools to evaluate the ROS level in the brain tissue.

### 2.6. Quantitative Reverse Transcription PCR (qRT-PCR)

Total RNA was extracted from brain tissue using the TRIZOL Kit (Accurate Biotech Co., Ltd., Changsha, China), as described in a previous study [23]. Real-time PCR was performed using the SYBR Green Master Mix (Accurate Biotech Co., Ltd., Changsha, China). The specific procedure was carried out as outlined in a previous study. To normalize the results, β-actin was used as the reference gene, and the expression levels of the target genes were calculated using the 2^−ΔΔCT^. The primer of genes was listed in Supplementary Table S2.

### 2.7. Western Blotting

Total protein samples were extracted from brain tissue, followed by separation using sodium dodecyl sulfate (SDS)-polyacrylamide gel electrophoresis. The proteins were then transferred to a polyvinylidene fluoride (PVDF) membrane. Membranes were blocked with 5% non-fat milk for 2 hours, then incubated overnight at 4 °C with primary antibodies (Occludin, Claudin-1, Nrf2, HO-1, β-actin, NLRP3, caspase-1, and ASC). After washing, membranes were incubated with HRP-conjugated secondary antibodies for 1 hour at room temperature. The electrochemiluminescence detection system (Image LabTM Software Version 3.0) was used for protein detection. ImageJ software (Version 1.52v, National Institutes of Health, Bethesda, MD, USA) was used to quantify relative protein expression.

### 2.8. Morris Water Maze Test for Cognitive Function

To explore the protective effect of BA on T-2 toxin-induced neuroinflammation in mice, the Morris water maze test was performed to evaluate cognitive function. The Morris water maze experiment was conducted in a circular pool with a 100 cm diameter, filled with 15 cm of water and made opaque by adding powdered milk. During the training phase, a platform was submerged at the center of one quadrant. Each mouse was given 60 s to find the platform. If unsuccessful, the mouse was gently guided to the platform and allowed to rest for 15 s. The training phase continued for 5 days. The testing trial was conducted 24 h after the last training session, with the platform removed. Each mouse was allowed to swim for 60 s. A digital camera was used to monitor each mouse’s performance, recording its swimming path, the number of crossings of the platform location, and the time spent in the platform’s quadrant.

### 2.9. Network Database for T-2 Toxin Toxicity Analysis

In the toxicity analysis of T-2 toxin, a detailed structural analysis was conducted using the PubChem database (https://pubchem.ncbi.nlm.nih.gov (accessed on 2 December 2025)), establishing the standard chemical structure and SMILES notation of T-2 toxin. After that, we used ProTox 3.0 (https://tox.charite.de/protox3/ (accessed on 2 December 2025)), a virtual lab for predicting the toxicities of small molecules, to identify possible target sites of T-2 toxin. These resources identified potential targets associated with T-2 toxin’s exposure.

### 2.10. Network Pharmacological Analysis

Screening of common targets: Search for BA secondary structure in the PubChem database (https://pubchem.ncbi.nlm.nih.gov (accessed on 2 December 2025)) and import it into the Swiss Target Prediction database (http://www.swisstargetprediction.ch (accessed on 2 December 2025)) to obtain BA target and target information; enter “neuroinflammation” in the Gene Cards database (https://www.genecards.org (accessed on 2 December 2025)) to retrieve the target of neuroinflammation. Construction of the PPI network and screening of core targets: In the STRING database (https://string-db.org (accessed on 2 December 2025)), select “Multiple proteins”, enter the common target obtained above, select “Mus” as the species, and build the PPI network. Enter the node information from the file into Cytoscape 3.7.2 (https://cytoscape.org (accessed on 2 December 2025)) for the network diagram, and then use the network analyzer for topology analysis. The targets should be sorted according to the strength of protein association. Finally, select the core targets.

### 2.11. Molecular Docking of BA and T-2 Toxin with the Target Protein

Firstly, the crystal structure files of the protein target were downloaded from the PDB database (RCSB PDB: Homepage). Obtain the chemical structures of the compounds from the PubChem database. Then, use Autodock 4.2.6 for virtual docking, and the docking results were visually analyzed with PyMOL 2.2.0 and Discovery Studio Client v19.1.0.

### 2.12. Molecular Dynamics Simulations

Molecular dynamics was simulated using GROMACS 2022.3. Specific parameters were determined according to a previously reported method. After the completion of the simulation, the trajectories were analyzed using the tools of the software, and the root mean square deviation (RMSD), root mean square rise and fall (RMSF), hydrogen bonds (HBond), solvent-accessible surface area (SASA), protein radius of gyration (Rg), and the free binding energy (MM-PBSA) of each amino acid trajectory were calculated.

### 2.13. Statistical Analysis

All statistical analyses were performed using SPSS 25.0. After verifying normality and homogeneity of variance using the Shapiro–Wilk and Levene’s tests, data were analyzed using one-way analysis of variance (ANOVA) followed by LSD post hoc tests. All statistical analyses were conducted using GraphPad Prism 8.0, and data are presented as the mean ± standard error of the mean. A *p*-value of < 0.05 was considered statistically significant, and a *p*-value of < 0.01 indicated high significance.

## 3. Results

### 3.1. T-2 Toxin Induces Neuroinflammation in Mice

**Figure 1:**
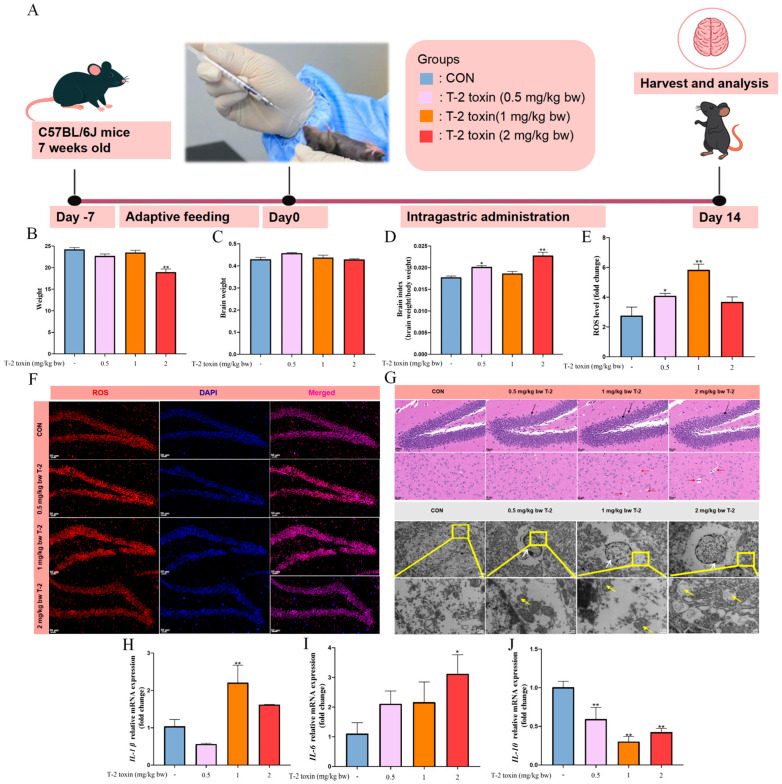
Effects of T-2 toxin on neuroinflammation in mice. (**A**) Schematic representation of the experimental design. (**B**) Body weight. (**C**) Brain weight. (**D**) Brain index. (**E**,**F**) The content of ROS. (**G**) H&E staining revealed brain morphological structures (black arrows: cell disruption and lysis, red arrows: bleeding visible, scale bar: 20 μm). TEM visualized ultrastructural changes (white arrows: nuclei invagination, yellow arrows: mitochondrial crest reduction, swelling, and vacuolation, scale bars: 5 μm and 2 μm). (**H**–**J**) qRT-PCR was used to measure *IL-1β*, *IL-6,* and *IL-10* mRNA levels in the brain of mice. * *p* < 0.05, ** *p* < 0.01 as compared to the CON group.

To explore the effect of T-2 toxin exposure on neuroinflammation, the mice were administered T-2 toxin at 0.5, 1, or 2 mg/kg/bw for two weeks (Figure 1A). As shown in Figure 1B–D, body weight was significantly lower in the 2 mg/kg/bw group than in the CON group. Meanwhile, the brain index was higher in the 0.5 and 2 mg/kg/bw groups than in the CON group, with no change in brain weight. Additionally, exposure to 1 mg/kg/bw of T-2 toxin significantly increased ROS accumulation in the brains of mice compared with the CON group (*p* < 0.05) (Figure 1E,F). Histological examination showed greater neuronal dispersion and cortical hemorrhage, most evident at 1 mg/kg/bw. TEM revealed nuclear membrane invagination, reduced mitochondrial cristae, and mitochondrial disorganization across all T-2 toxin groups, whereas marked mitochondrial vacuolization was observed only in the 2 mg/kg/bw group (Figure 1G). qRT-PCR analysis showed that *IL-6* mRNA levels were elevated at 2 mg/kg/bw in the T-2 toxin group relative to the CON group. Compared with the CON group, *IL-1β* mRNA expression was significantly increased in the 1 mg/kg/bw group (*p* < 0.05). Moreover, *IL-10* mRNA levels were markedly decreased in the 1 and 2 mg/kg/bw groups (Figure 1H–J). Based on these findings, the dose of 1 mg/kg/bw produced the most robust oxidative and neuroinflammatory response and was selected for subsequent experiments.

### 3.2. T-2 Toxin Induces the Blood–Brain Barrier Injury in Mice

**Figure 2:**
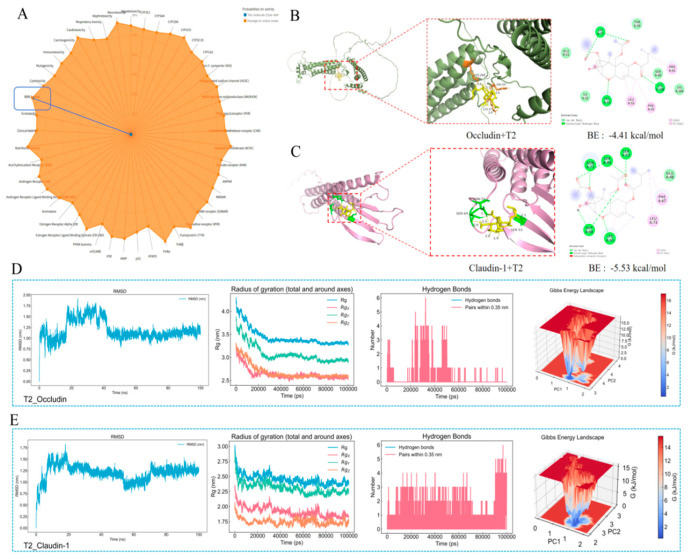
Effects of T-2 toxin on the blood–brain barrier in Mice. (**A**) The toxicity radar chart for T-2 toxin. (**B**,**C**) Molecular docking results of T-2 toxin with Occludin and Claudin-1, respectively. (**D**) MD simulation results of the Occludin and T-2 toxin complex, including RMSD, radius of gyration, hydrogen bond analysis, and the free energy landscape of the protein–ligand complex, respectively. (**E**) Similar analysis for the molecular dynamics results of T-2 toxin with Claudin-1.

To identify the molecular targets implicated in T-2 toxin–induced neuroinflammation, we performed Pro Tox 3.0 analysis, which predicted that T-2 toxin primarily affects the blood–brain barrier. (Figure 2A). Molecular docking showed that T-2 toxin binds to the tight junction proteins Occludin and Claudin-1, with docking energies of −4.41 and −5.53 kcal/mol, respectively (Figure 2B,C), indicating that T-2 toxin has a strong binding affinity for Occludin and Claudin-1. Molecular dynamics (MD) simulations further evaluated the stability of these interactions. The root-mean-square deviation (RMSD) of the T-2 toxin–Occludin protein–ligand complex stabilized after 40 ns, suggesting structural equilibrium. The radius of gyration (Rg) of Occludin decreased and stabilized after 20 ns, indicating a more compact conformation upon ligand binding. The number of hydrogen bonds also stabilized after 60 ns, confirming the formation of a stable protein–ligand complex. Principal component analysis (PCA) revealed that the complex occupied a stable, low-energy conformational region (Figure 2D). Similar MD simulation results were observed for the T-2 toxin–Claudin-1 complex (Figure 2E). Collectively, these findings demonstrate that T-2 toxin can interact stably with tight junction proteins, suggesting that it may compromise blood–brain barrier integrity and contribute to neuroinflammation.

### 3.3. BA Attenuates T-2 Toxin-Induced Brain Damage, Cognitive Impairment, and Neuroinflammation in Mice

**Figure 3:**
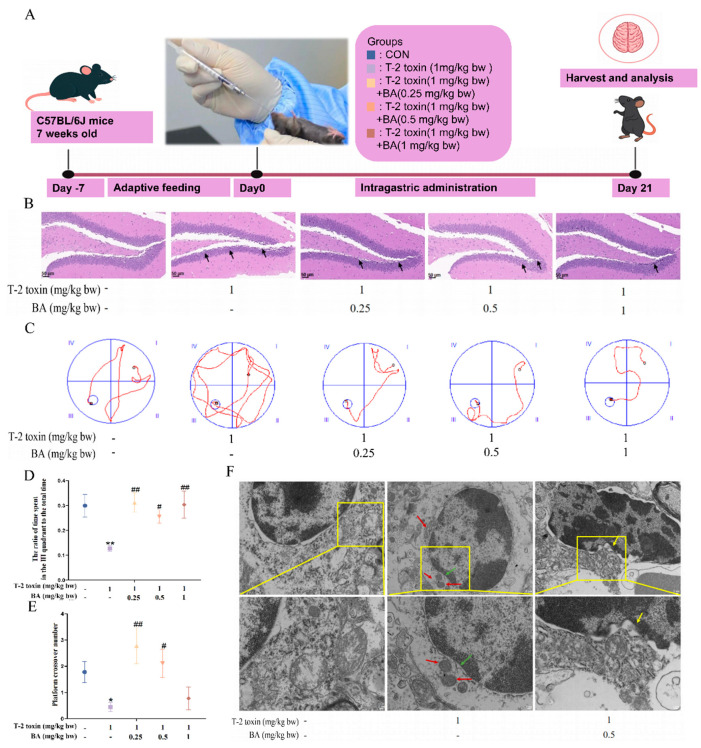
Effects of BA on T-2 toxin-induced brain damage and cognitive deficits in mice. (**A**) Schematic representation of the experimental design. (**B**) H&E staining observation of brain morphology and structure, autopsy (black arrow: disordered arrangement of cells and disrupted cell morphology, scale bar: 50 μm). (**C**) Trajectory plot of mice arriving at the platform. (**D**) Time spent by mice in quadrant III. (**E**) Number of platform crossings by mice. (**F**) Observe the ultrastructure by TEM (red arrow: nuclear invagination, green arrow: mitochondrial swelling and vacuolization, yellow arrow: rupture of endoplasmic reticulum, scale bars: 2 μm and 1 μm). * *p* < 0.05, ** *p* < 0.01 as compared to the CON group. # *p* < 0.05, ## *p* < 0.01 as compared to the T-2 toxin group.

We next investigated whether BA could alleviate T-2 toxin–induced neuroinflammation in mice (Figure 3A). H&E staining revealed that BA markedly attenuated the irregular cellular boundaries and nuclear deformation or rupture in hippocampal dentate gyrus neurons induced by T-2 toxin exposure (Figure 3B). At the ultrastructural level, hippocampal dentate gyrus neurons from T-2 toxin–treated mice exhibited pronounced nuclear membrane invagination, mitochondrial swelling, and cytoplasmic vacuolization. In contrast, these pathological alterations were substantially ameliorated in the BA-treated groups (Figure 3F). Cognitive function was further evaluated using the Morris water maze test. Mice in the T-2 toxin group showed reduced time spent in the target quadrant and fewer platform crossings than those in the CON group. In contrast, BA administration markedly improved these effects (*p* < 0.05) (Figure 3C–E).

### 3.4. BA Attenuates T-2 Toxin-Induced Oxidative Stress and Neuroinflammation in Mice

**Figure 4:**
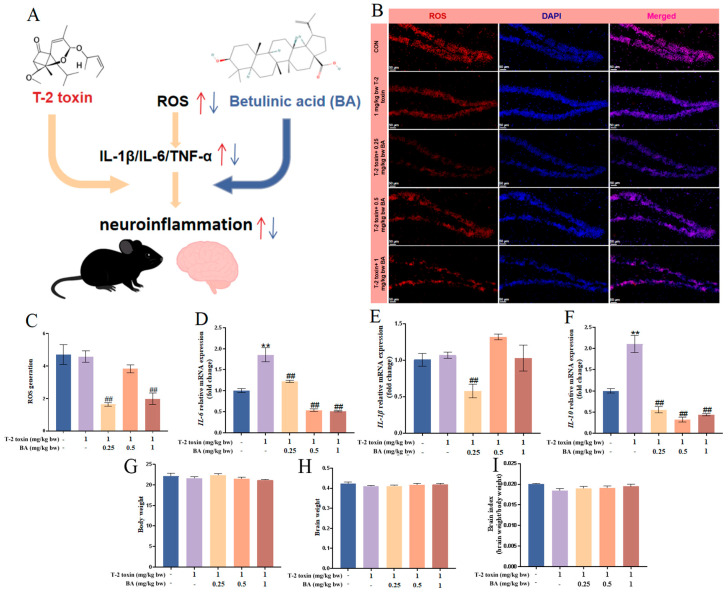
Effects of BA on T-2 toxin-induced Oxidative stress and neuroinflammation in mice. (**A**) Graphical Abstract. (**B**,**C**) The content of ROS. (**D**–**F**) qRT-PCR was used to measure *IL-6*, *IL-1β*, and *IL-10* mRNA levels in the brain. (**G**) Body weight. (**H**) Brain weight. (**I**) Brain index. ** *p* < 0.01 as compared to the CON group. ## *p* < 0.01 as compared to the T-2 toxin group.

In addition, oxidative stress and inflammatory responses in mouse brain tissue were assessed to further characterize the effects of BA. Compared with the T-2 toxin-treated group, the ROS level was significantly reduced by 0.25 and 1 mg/kg/bw BA, whereas 0.5 mg/kg/bw BA exhibited a non-significant downward trend (*p* < 0.05) (Figure 4B,C). qRT-PCR analysis showed that T-2 toxin administration markedly increased the mRNA levels of *IL-6*, *IL-10* and compared with the CON group (Figure 4D–F). However, BA treatment significantly decreased the mRNA levels of *IL-6* and *IL-10* compared to the T-2 toxin-treated group (Figure 4D–F). In addition, there were no statistically significant differences in body weight, brain weight, or brain index between any treatment group and the CON group (Figure 4G–I). Together, these results demonstrated that BA effectively alleviated T-2 toxin-induced oxidative stress and neuroinflammation in the brain of mice.

### 3.5. BA Alleviates T-2 Toxin-Induced Blood–Brain Barrier Damage

**Figure 5:**
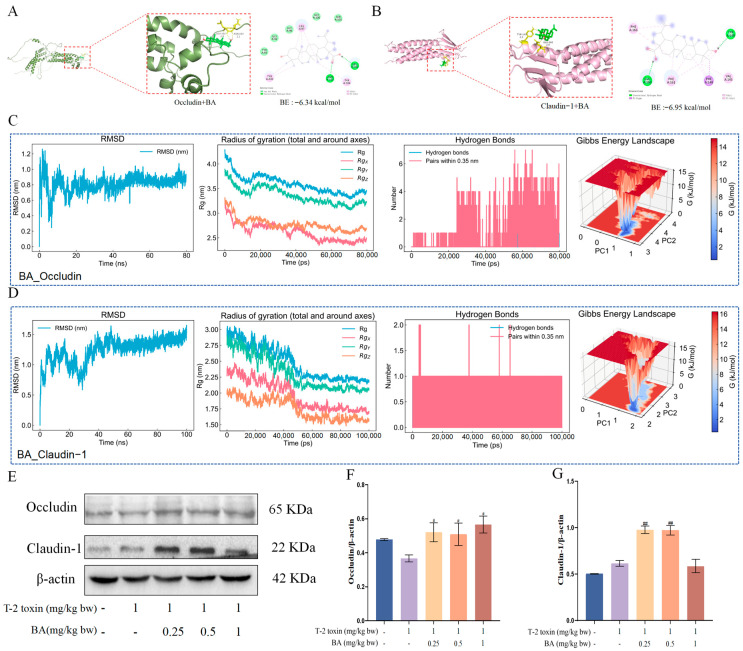
Effect of BA on T-2 toxin-induced blood–brain barrier in mice. (**A**,**B**) Molecular docking results between BA and Occludin and Claudin-1, respectively. (**C**,**D**) MD simulation results of the Occludin and BA, Claudin-1 and BA, including RMSD, radius of gyration, hydrogen bond analysis, and the free energy landscape of the protein–ligand complex. (**E**–**G**) Western blot was used to detect protein expression of Occludin, Claudin-1, and β-actin. The original Western blot images are provided in the Supplementary Materials. # *p* < 0.05, ## *p* < 0.01 as compared to the T-2 toxin group.

To investigate the protective mechanism of BA against T-2 toxin-induced neuroinflammation, molecular docking analysis revealed strong binding affinity of BA to Occludin and Claudin-1, with binding energies of −6.34 and −6.95 kcal/mol, respectively (Figure 5A,B). MD simulations confirmed the stability of the interactions. RMSD of the BA–Occludin protein–ligand complex stabilized after 50 ns, while the Rg of Occludin decreased and reached a stable plateau after 60 ns, suggesting a more compact protein conformation. The number of hydrogen bonds also stabilized after 70 ns, further supporting the formation of a stable complex. The PCA revealed that the complex occupied a stable, low-energy conformational region (Figure 5C). Similar patterns were observed in the BA–Claudin-1 protein–ligand complex (Figure 5D). As shown in Figure 5E,F, compared with the T-2 toxin-treated group, the protein levels of Occludin and Claudin-1 were markedly increased in the BA-treated groups. Collectively, these findings suggest that BA could alleviate T-2 toxin–induced blood–brain barrier injury.

### 3.6. BA Alleviates T-2 Toxin-Induced Neuroinflammation by Modulating the Nrf2/NLRP3 Signaling Pathway in Mice

**Figure 6:**
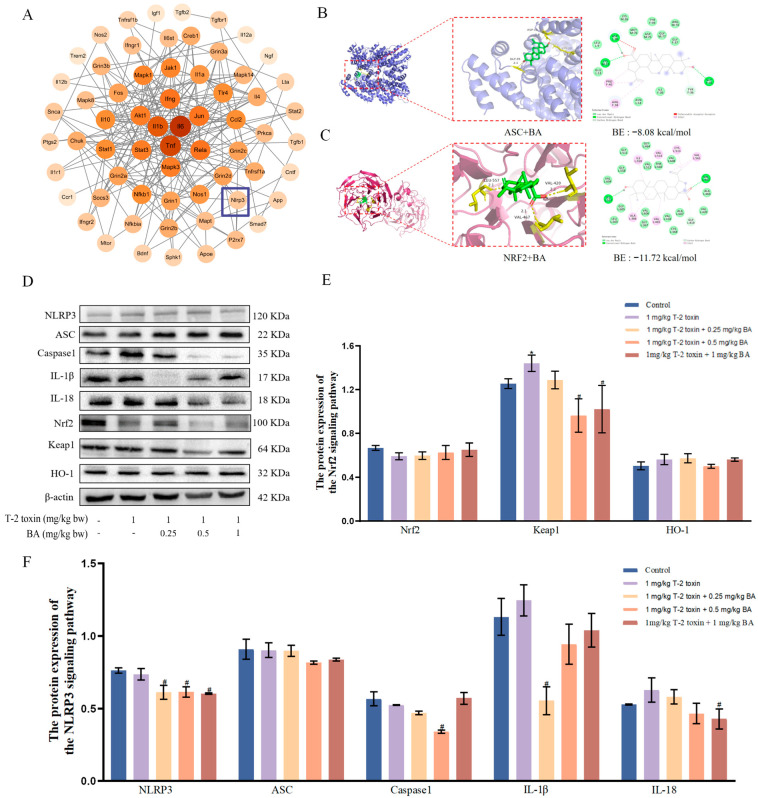
Effect of BA on the Nrf2/NLRP3 signaling pathway by T-2 toxin exposure in mice. (**A**) Protein network interactions in neuroinflammation. (**B**,**C**) Molecular docking results between BA and ASC and Nrf2, respectively. (**D**–**F**) Western blot was used to detect protein expression of Nrf2, Keap1, HO-1, NLRP3, ASC, Caspase-1, IL-1β, IL-18, and β-actin in the brain of mice. * *p* < 0.05 as compared to the CON group. # *p* < 0.05 as compared to the T-2 toxin group.

To investigate the mechanisms by which BA alleviated T-2 toxin-induced neuroinflammation, a protein–protein interaction (PPI) network associated with neuroinflammation was constructed. As shown in the network map, core inflammation- and immune-related proteins, including IL-6 and IL-1β, were positioned centrally. Although NLRP3 was located at the periphery, its interacting proteins and the surrounding region were associated with inflammatory signaling pathways, suggesting potential involvement of the NLRP3 inflammasome-related pathway in the network (Figure 6A). Molecular docking analysis showed that BA exhibited strong binding affinity to ASC and Nrf2, with binding energies of −6.34 kcal/mol and −6.95 kcal/mol, respectively (Figure 6B,C). Compared with the CON group, T-2 toxin induced a decreasing trend in Nrf2 protein expression and an increase in Keap1 protein expression, while BA pretreatment reversed these alterations. No significant differences in HO-1 protein expression were observed among all groups. Furthermore, compared with the T-2 toxin-treated group, BA pretreatment downregulated the protein expression of NLRP3, Caspase-1, ASC, IL-1β, and IL-18(Figure 6D–F). Collectively, BA mitigated T-2 toxin-induced neuroinflammation through activation of the Nrf2 signaling pathway and inhibition of the NLRP3 signaling pathway in mice.

## 4. Discussion

T-2 toxin is a highly prevalent trichothecene mycotoxin that commonly contaminates agricultural products and animal feed, posing a serious threat to food safety and public health [24]. Increasing evidence indicates that T-2 toxin exerts pronounced neurotoxicity, characterized by oxidative stress, neuroinflammation, and disruption of central nervous system homeostasis [25]. Consistent with previous reports, T-2 toxin triggered excessive ROS accumulation and activated inflammatory cascades in the central nervous system, consistent with the neurotoxic mechanism induced by other trichothecene mycotoxins [26]. BA is a naturally occurring pentacyclic triterpenoid primarily derived from the bark of birch trees and other medicinal plants [10]. Our previous studies had shown that BA possesses potent anti-inflammatory and antioxidant properties, effectively mitigating mycotoxin-induced inflammatory responses and oxidative damage [20,22]. However, whether BA can protect against T-2 toxin-induced neuroinflammation and the underlying molecular mechanisms remain unclear. In this study, we demonstrated that BA markedly alleviated T-2 toxin-induced neuroinflammation and blood–brain barrier injury by regulating the Nrf2/NLRP3 signaling pathway in mice.

It has been reported that T-2 toxin has many toxic effects, including immunotoxicity, reproductive toxicity, hepatotoxicity, neurotoxicity, cardiotoxicity, and bone system damage [27]. However, the specific effect and underlying mechanisms of T-2 toxin in the central nervous system, especially in relation to blood–brain barrier integrity and neuroinflammatory signaling, remain largely unexplored. Damage to the central nervous system is a key manifestation of T-2 toxin-induced toxicity. Previous studies found that 4 mg/kg/bw of T-2 toxin exposure significantly induced cortical inflammatory infiltration, nuclear and mitochondrial damage, elevated ROS levels, and increased inflammatory cytokine secretion in the brains of mice [6]. In addition, in vivo studies have further suggested that T-2 toxin might compromise blood–brain barrier permeability and elevate inflammatory cytokine expression, supporting the involvement of barrier dysfunction in its neurotoxic effects [28]. Moreover, T-2 toxin and its major metabolite HT-2 were shown to disrupt blood–brain barrier integrity [29]. In this study, T-2 exposure leads to marked neuronal structural damage, including neuronal atrophy, cell swelling, pericellular space enlargement, cortical hemorrhage, and increased neuronal apoptosis. In addition, our data showed that 1 mg/kg/bw T-2 toxin exposure elicited the most pronounced oxidative stress and neuroinflammatory response, characterized by increased levels of ROS and pro-inflammatory cytokines. Meanwhile, T-2 toxin exposure led to neuronal dispersion and marked ultrastructural damage, including nuclear membrane invagination and disruption of mitochondrial cristae. In addition, T-2 toxin exhibited binding affinity toward the Occludin and Claudin-1 proteins, with binding energies of −4.41 and −5.53 kcal/mol, respectively, which inferred that we successfully established a T-2 toxin induced neuroinflammation model in mice and identify 1 mg/kg/bw T-2 toxin as the optimal dose for subsequent experiments. These results are in line with previous studies showing that BA improved cognitive function in multiple brain injury models by suppressing oxidative stress and neuronal damage [30].

In recent years, accumulating evidence indicates that plant-derived phytochemicals exert protective effects on blood–brain barrier integrity and on neuroinflammatory responses. For example, resveratrol has been reported to reduce blood–brain barrier permeability and preserve tight junction proteins under inflammatory or ischemic conditions [31]. Curcumin could mitigate blood–brain barrier disruption by inhibiting oxidative stress and pro-inflammatory signaling [32]. Taken together, these findings suggested that plant-derived agents can stabilize the neurovascular unit by targeting redox imbalance and inflammation. BA is a pentacyclic triterpenoid abundantly present in birch bark and other woody plants, with well-documented antioxidant, anti-inflammatory, antitumor, and immunomodulatory properties. Accumulating evidence suggested that BA exerts neuroprotective effects in experimental models of brain injury triggered by various injurious factors, largely through its antioxidant and anti-inflammatory properties [33]. However, its effects on T-2 toxin-induced brain injury remain unclear. In our study, we found that BA markedly ameliorated T-2 toxin exposure-induced brain injury by reducing neuronal disorganization, nuclear deformation, mitochondrial swelling, and vacuolization in the hippocampal dentate gyrus. Meanwhile, BA clearly alleviated T-2 toxin-induced cognitive dysfunction, characterized by simpler swimming paths, increased time spent in the target quadrant, and more frequent platform crossings. In addition, BA could reduce ROS accumulation induced by T-2 toxin in the brains of mice. Although the optimal doses for ROS reduction and inflammasome inhibition were not entirely identical, all BA doses shifted the redox state toward a less oxidative condition. Molecular docking analysis showed that BA binds Occludin or Claudin-1 with binding energies of −6.34 or −6.95 kcal/mol, respectively. Consistently, molecular dynamics simulations revealed that BA has relatively strong affinities to Occludin and Claudin-1. Furthermore, BA could increase the blood–brain barrier-related protein levels induced by T-2 toxin exposure. This effect is analogous to that of other neuroprotective plant-derived compounds, such as resveratrol and curcumin, which preserve BBB integrity by stabilizing tight junction proteins under neuroinflammatory conditions [34]. Taken together, BA could attenuate T-2 toxin-induced neurotoxicity by alleviating hippocampal structural and mitochondrial damage, suppressing oxidative stress and inflammatory response, and improving spatial learning and memory in mice. These results are consistent with previous studies showing that BA improved cognitive function across multiple brain injury models by suppressing oxidative stress and neuronal damage.

To investigate the mechanisms by which BA alleviated T-2 toxin-induced neuroinflammation, a neuroinflammation-related protein interaction network was constructed, in which IL-6, IL-1β occupied central positions, whereas NLRP3 was located at the periphery but remained functionally linked to key inflammatory signaling nodes. Consistent with this network topology, BA treatment was accompanied by reduced IL-6 and TNF-α mRNA expression, indicating that BA mitigated oxidative stress-driven neuroinflammation. Accumulating evidence has demonstrated that neuroinflammation represents a critical pathological process in mycotoxin-induced brain injury. Excessive production of pro-inflammatory cytokines, microglial activation, and disruption of blood–brain barrier integrity collectively contribute to neuronal dysfunction and brain damage after mycotoxin exposure [35]. Importantly, oxidative stress is increasingly recognized as an upstream driver of neuroinflammatory cascades, providing a mechanistic link between redox imbalance and sustained inflammatory activation in the central nervous system [13]. However, it is not clear whether the BA alleviation of T-2 toxin-induced neuroinflammation was related to oxidative stress. Based on these considerations, we further focused on the Nrf2/NLRP3 signaling pathway as a critical regulatory axis connecting oxidative stress and inflammatory responses. Increasing evidence suggests that impaired Nrf2 signaling facilitates ROS accumulation, thereby lowering the NLRP3 inflammasome activation threshold and promoting sustained inflammatory signaling in the brain [36]. Accordingly, the Nrf2/NLRP3 axis has emerged as a critical molecular link between oxidative stress and neuroinflammation in various models of brain injury. Previous studies have reported that T-2 toxin induces oxidative stress by disrupting the Nrf2/Keap1 regulatory system, resulting in redox imbalance [37]. Meanwhile, impairment of Nrf2-mediated antioxidant defenses may create a permissive intracellular environment for the NLRP3 inflammasome activation, thereby amplifying neuroinflammatory injury [38]. Hence, restoration of Nrf2 signaling and suppression of NLRP3 inflammasome activation represent a mechanistically plausible strategy for attenuating T-2 toxin-induced oxidative stress and neuroinflammation. Consistent with our findings, BA pretreatment effectively restored the balance of Nrf2/Keap1 signaling by upregulating Nrf2 protein expression and markedly reducing Keap1 levels induced by T-2 toxin exposure, indicating reactivation of Nrf2-mediated antioxidant defense. Although heme HO-1 protein expression remained unchanged among all groups, this suggested that BA primarily acts at the upstream regulatory level of the Nrf2/Keap1 axis or through other Nrf2-dependent antioxidant targets. Furthermore, BA administration downregulated NLRP3 protein expression across all tested doses and most effectively suppressed Caspase-1 activation at 0.5 mg/kg/bw, accompanied by a tendency to decrease ASC protein expression, reflecting a dose-dependent inhibition of inflammasome activation. These findings are consistent with studies demonstrating that Nrf2 activation negatively regulates the NLRP3 inflammasome in neuroinflammatory diseases [38]. In parallel, BA treatment notably reduced the production of pro-inflammatory cytokines induced by T-2 toxin exposure in the brains of mice. Consistent with these findings, molecular docking analysis revealed that BA exhibited strong binding affinities to ASC and Nrf2, with binding energies of −8.08 kcal/mol and −11.72 kcal/mol, respectively, suggesting stable interactions that may underlie its dual regulatory effects on oxidative stress and inflammasome activation. Our results are supported by previous reports showing that BA attenuated inflammation and oxidative stress via Nrf2-dependent pathways in LPS-, cyclophosphamide-, and zearalenone-induced models [20]. Taken together, these data indicated that BA mitigated T-2 toxin–induced neuroinflammation by activating the Nrf2/Keap1 signaling pathway and inhibiting NLRP3 inflammasome activation. This study provides the first direct evidence that BA attenuated T-2 toxin-induced neuroinflammation, blood–brain barrier impairment, and cognitive dysfunction by modulating the Nrf2/NLRP3 signaling axis. In contrast to previous studies that mainly focused on the peripheral organ toxicity of T-2 toxin, the present work reveals a key mechanism by which oxidative stress and inflammatory signaling mediate blood–brain barrier disruption and neuronal damage. These findings extend the neuroprotective spectrum of BA and establish a new natural candidate for counteracting mycotoxin-associated neurotoxicity. Considering the widespread contamination of T-2 toxin in agricultural products and feeds, these results offer a promising nutritional and pharmacological strategy for preventing and treating neurotoxic disorders caused by mycotoxin exposure in both animals and humans.

Despite the mechanistic insights this study provides, several limitations warrant consideration. Although in vivo and in silico analyses implicated the Nrf2/NLRP3 axis in T-2 toxin-induced neuroinflammation, a direct causal relationship was not established using genetic or pharmacological loss-of-function approaches. In addition, predicted interactions between T-2 toxin or BA and barrier- or inflammasome-related proteins derived from computational analyses require experimental validation. The present study focused on acute or subacute exposure. However, the long-term effects of chronic low-dose T-2 toxin exposure and the protective effects of BA on sustained neuroprotection remain to be determined. Future studies should further explore the Nrf2/NLRP3 axis as a therapeutic target for mycotoxin-associated neurotoxicity.

## 5. Conclusions

Our study demonstrated that T-2 toxin-driven oxidative stress disrupted blood–brain barrier integrity and promoted neuroinflammation. However, BA could alleviate T-2 toxin-induced neuroinflammation, oxidative stress, blood–brain barrier injury, and cognitive disorder through regulating the Nrf2/NLRP3 signaling pathway. This study provides a novel theoretical foundation for developing nutritional or pharmacological interventions against mycotoxin-related neurological disorders. Moreover, this study has several limitations. All experiments were performed in vivo, without in vitro validation or clinical translational data. Genetic or pharmacological intervention targeting the Nrf2/NLRP3 axis was not performed. Hence, the direct causal link remains to be confirmed. The long-term safety and optimal dosage of BA require further evaluation. Collectively, our findings suggest that BA represents a promising natural candidate against mycotoxin-associated neurological disorders, and further research is needed to promote its translational application.

## Data Availability

The original contributions presented in this study are included in the article’s Supplementary Material. Further inquiries can be directed to the corresponding author.

## Supplementary Materials

The following supporting information can be downloaded at https://www.mdpi.com/article/10.3390/vetsci13060509/s1. Table S1: Main reagents; Table S2: Primer sequences. Figures: Original Western blot images.

## References

[1] Xu C., Oliveri Conti G., Tang S., Shen J., Dai C. (2026). T-2 Toxin Neurotoxicity: Molecular Mechanisms and Emerging Chemoprotective Strategies. Antioxidants.

[2] He W., Zhu Z., Xu J., Huang C., Wang J., Wang Q., Zhai X., Yang J. (2025). Integrated Cytotoxicity and Metabolomics Analysis Reveals Cell-Type-Specific Responses to Co-Exposure of T-2 and HT-2 Toxins. Toxins.

[3] Sun T., Zhang Q., Li M., Tang S., Dai C. (2022). T-2 Toxin Induces Apoptotic Cell Death and Protective Autophagy in Mouse Microglia BV2 Cells. J. Fungi.

[4] Fernye C., Ancsin Z., Bócsai A., Balogh K., Mézes M., Erdélyi M. (2018). Role of Glutathione Redox System on the T-2 Toxin Tolerance of Pheasant (*Phasianus colchicus*). Toxicol. Res..

[5] Gonkowski S., Gajęcka M., Makowska K. (2020). Mycotoxins and the Enteric Nervous System. Toxins.

[6] Kuć-Szymanek A., Kubik-Machura D., Kościelecka K., Męcik-Kronenberg T., Radko L. (2025). Neurotoxicological Effects of Some Mycotoxins on Humans Health and Methods of Neuroprotection. Toxins.

[7] Paes A.S., Koga R.d.C.R., Sales P.F., Santos Almeida H.K., Teixeira T.A.C.C., Carvalho J.C.T. (2023). Phytocompounds from Amazonian Plant Species against Acute Kidney Injury: Potential Nephroprotective Effects. Molecules.

[8] Chen J., Huang Z., Cao X., Chen X., Zou T., You J. (2022). Plant-Derived Polyphenols as Nrf2 Activators to Counteract Oxidative Stress and Intestinal Toxicity Induced by Deoxynivalenol in Swine: An Emerging Research Direction. Antioxidants.

[9] Wang P., Sun L.-H., Wang X., Wu Q., Liu A. (2024). Effective Protective Agents against the Organ Toxicity of T-2 Toxin and Corresponding Detoxification Mechanisms: A Narrative Review. Anim. Nutr..

[10] Wang S., Zhang Y., Yang X., Wang K., Yang X., Zhang B., Zhang B., Bie Q. (2024). Betulinic Acid Arrests Cell Cycle at G2/M Phase by up-Regulating Metallothionein 1G Inhibiting Proliferation of Colon Cancer Cells. Heliyon.

[11] An K., Shi B., Lv X., Liu Y., Xia Z. (2024). T-2 Toxin Triggers Lipid Metabolism Disorder and Oxidative Stress in Liver of Ducks. Ecotoxicol. Environ. Saf..

[12] Dai C., Xiao X., Sun F., Zhang Y., Hoyer D., Shen J., Tang S., Velkov T. (2019). T-2 Toxin Neurotoxicity: Role of Oxidative Stress and Mitochondrial Dysfunction. Arch. Toxicol..

[13] Ridolfi E., Barone C., Scarpini E., Galimberti D. (2013). The Role of the Innate Immune System in Alzheimer’s Disease and Frontotemporal Lobar Degeneration: An Eye on Microglia. J. Immunol. Res..

[14] Taroncher M., Franco-Campos F., Rodríguez-Carrasco Y., Ruiz M.-J. (2025). T-2 Toxin-Induced Hepatotoxicity in HepG2 Cells Involves the Inflammatory and Nrf2/HO-1 Pathways. Toxins.

[15] Costa R.M., Dias M.C., Alves J.V., Silva J.L.M., Rodrigues D., Silva J.F., Francescato H.D.C., Ramalho L.N.Z., Coimbra T.M., Tostes R.C. (2024). Pharmacological Activation of Nuclear Factor Erythroid 2-Related Factor-2 Prevents Hyperglycemia-Induced Renal Oxidative Damage: Possible Involvement of O-GlcNAcylation. Biochem. Pharmacol..

[16] Lu R., Zhou X., Zhang L., Hao M., Yang X. (2024). Nrf2 Deficiency Exacerbates Parkinson’s Disease by Aggravating NLRP3 Inflammasome Activation in MPTP-Induced Mouse Models and LPS-Induced BV2 Cells. J. Inflamm. Res..

[17] Uruno A., Yamamoto M. (2023). The KEAP1-NRF2 System and Neurodegenerative Diseases. Antioxid. Redox Signal..

[18] Ou Z., Zhu L., Huang C., Ma C., Kong L., Lin X., Gao X., Huang L., Wen L., Liang Z. (2021). Betulinic Acid Attenuates Cyclophosphamide-Induced Intestinal Mucosa Injury by Inhibiting the NF-κB/MAPK Signalling Pathways and Activating the Nrf2 Signalling Pathway. Ecotoxicol. Environ. Saf..

[19] Zhu L., Kong L., Huang Y., Ou Z., Huang C., Yang W., He J., Yang M., Liu S., Yi J. (2025). Betulinic Acid Protects against LPS-Induced Intestinal Inflammatory Damage via Inhibiting Nrf2/TXNIP/NLRP3 Signaling Pathways in Mice. Food Funct..

[20] Wu J., Li J., Wu Y., Yang M., Chen Y., Wang N., Wang J., Yuan Z., Yi J., Yang C. (2023). Betulinic Acid Mitigates Zearalenone-Induced Liver Injury by ERS/MAPK/Nrf2 Signaling Pathways in Mice. Food Chem. Toxicol..

[21] Wu C., Chen H., Zhuang R., Zhang H., Wang Y., Hu X., Xu Y., Li J., Li Y., Wang X. (2021). Betulinic Acid Inhibits Pyroptosis in Spinal Cord Injury by Augmenting Autophagy via the AMPK-mTOR-TFEB Signaling Pathway. Int. J. Biol. Sci..

[22] Huang Y., Zhu Z., Luo C., Ma C., Zhu L., Kong L., Li R., Wu J., Yuan Z., Yi J. (2022). Betulinic Acid Attenuates Cognitive Dysfunction, Oxidative Stress, and Inflammation in a Model of T-2 Toxin-Induced Brain Damage. Environ. Sci. Pollut. Res..

[23] Zhao H., Huang Y., Yang W., Huang C., Ou Z., He J., Yang M., Wu J., Yao H., Yang Y. (2024). *Viola Yedoensis* Makino Alleviates Lipopolysaccharide-Induced Intestinal Oxidative Stress and Inflammatory Response by Regulating the Gut Microbiota and NF-κB-NLRP3/ Nrf2-MAPK Signaling Pathway in Broiler. Ecotoxicol. Environ. Saf..

[24] Wang K., Perveen A., Shen J., Kaka N.A., Li Y., Shen D., Li C. (2026). Maternal T-2 Mycotoxin Exposure Impairs Liver Function and Growth in Both Dams and Offspring in Mice. J. Appl. Toxicol..

[25] Wang Y., Wang B., Wang P., Hua Z., Zhang S., Wang X., Yang X., Zhang C. (2024). Review of Neurotoxicity of T-2 Toxin. Mycotoxin Res..

[26] Wang Y., Liu P., Fan J., Li S., Zhang X., Li Y., Wang X., Zhang C., Yang X. (2025). T-2 Toxin Nephrotoxicity: Toxic Effects, Mechanisms, Mitigations, and Future Perspectives. J. Agric. Food Chem..

[27] An K., Yan D., Lv X., Liu Y., Xia Z. (2025). T-2 Toxin Induces Gut and Liver Injury through Triggering Gut Microbiota Dysbiosis. Poult. Sci..

[28] Ravindran J., Agrawal M., Gupta N., Rao P.V.L. (2011). Alteration of Blood Brain Barrier Permeability by T-2 Toxin: Role of MMP-9 and Inflammatory Cytokines. Toxicology.

[29] Weidner M., Hüwel S., Ebert F., Schwerdtle T., Galla H.-J., Humpf H.-U. (2013). Influence of T-2 and HT-2 Toxin on the Blood-Brain Barrier in Vitro: New Experimental Hints for Neurotoxic Effects. PLoS ONE.

[30] Farzan M., Farzan M., Shahrani M., Navabi S.P., Vardanjani H.R., Amini-Khoei H., Shabani S. (2024). Neuroprotective Properties of Betulin, Betulinic Acid, and Ursolic Acid as Triterpenoids Derivatives: A Comprehensive Review of Mechanistic Studies. Nutr. Neurosci..

[31] Wei H., Wang S., Zhen L., Yang Q., Wu Z., Lei X., Lv J., Xiong L., Xue R. (2015). Resveratrol Attenuates the Blood-Brain Barrier Dysfunction by Regulation of the MMP-9/TIMP-1 Balance after Cerebral Ischemia Reperfusion in Rats. J. Mol. Neurosci..

[32] Yang Q., Li R., Hong Y., Liu H., Jian C., Zhao S. (2024). Curcumin-Loaded Gelatin Nanoparticles Cross the Blood-Brain Barrier to Treat Ischemic Stroke by Attenuating Oxidative Stress and Neuroinflammation. Int. J. Nanomed..

[33] Pandey S.K., Nanda A., Gautam A.S., Singh R.K. (2025). Betulinic Acid Protects against Lipopolysaccharide and Ferrous Sulfate-Induced Oxidative Stress, Ferroptosis, Apoptosis, and Neuroinflammation Signaling Relevant to Parkinson’s Disease. Free Radic. Biol. Med..

[34] Liu X., Baxley S., Hebron M., Turner R.S., Moussa C. (2025). Resveratrol Attenuates CSF Markers of Neurodegeneration and Neuroinflammation in Individuals with Alzheimer’s Disease. Int. J. Mol. Sci..

[35] Song C., Wang Z., Cao J., Dong Y., Chen Y. (2024). Neurotoxic Mechanisms of Mycotoxins: Focus on Aflatoxin B1 and T-2 Toxin. Env. Pollut..

[36] Ahmed S.M.U., Luo L., Namani A., Wang X.J., Tang X. (2017). Nrf2 Signaling Pathway: Pivotal Roles in Inflammation. Biochim. Biophys. Acta Mol. Basis Dis..

[37] Chen X., Mu P., Zhu L., Mao X., Chen S., Zhong H., Deng Y. (2021). T-2 Toxin Induces Oxidative Stress at Low Doses via Atf3ΔZip2a/2b-Mediated Ubiquitination and Degradation of Nrf2. Int. J. Mol. Sci..

[38] Tastan B., Arioz B.I., Genc S. (2022). Targeting NLRP3 Inflammasome With Nrf2 Inducers in Central Nervous System Disorders. Front. Immunol..

