# Supplementary material for: Betulinic Acid Ameliorates T-2 Toxin-Induced Neuroinflammation by Suppressing Oxidative Stress via Regulating Nrf2/NLRP3 Axis
Source: Vet Sci. 2026 May 24;13(6):509. doi: 10.3390/vetsci13060509 (PMC13308106; doi:10.3390/vetsci13060509)
Supplement: Supplementary file 1 [file vetsci-13-00509-s001.zip › Supplementary Tables.pdf]

Supplementary Table S1

Table S1 Main reagents

Reagent	Manufacturer
BA	Xi'an Beijinuo Biotechnology Co., Ltd., China
T-2 Toxin	Pribolab Pte Ltd., Singapore
4% Paraformaldehyde	Wuhan Google Biotechnology Co., Ltd., China
Hematoxylin Staining Solution	Wuhan Google Biotechnology Co., Ltd., China
Eosin Staining Solution	Wuhan Google Biotechnology Co., Ltd., China
Neutral Balsam Mounting Medium	Sinopharm Chemical Reagent Co., Ltd., China
Absolute Ethanol	Sinopharm Chemical Reagent Co., Ltd., China
Xylene	Sinopharm Chemical Reagent Co., Ltd., China
Electron Microscopy Fixative	Wuhan Google Biotechnology Co., Ltd., China
Evo M-MLV Reverse Transcription Kit	Hunan Acray Bioengineering Co., Ltd., Hunan, China
RNA Extraction Kit	Hunan Acray Bioengineering Co., Ltd., Hunan, China
SYBR Green Dye	Hunan Acray Bioengineering Co., Ltd., Hunan, China
Nrf2 Antibody	CST, Danvers, Massachusetts, USA
HO-1 Antibody	CST, Danvers, Massachusetts, USA
NLRP3 Antibody	CST, Danvers, Massachusetts, USA
Caspase-1 Antibody	CST, Danvers, Massachusetts, USA
ASC Antibody	CST, Danvers, Massachusetts, USA
IL-1β Antibody	CST, Danvers, Massachusetts, USA
IL-18 Antibody	Proteintech, Chicago, USA
β-actin Antibody	Proteintech, Chicago, USA
HRP-conjugated Goat Anti-Rabbit IgG	Proteintech, Chicago, USA

Supplementary Table S2

Table S2 Primer sequences

Gene Name	Primer Sequences (5' → 3')	Product Length	Accession Number
IL-1β	TGCCACCTTTTGACAGTGATG<br>TGATGTGCTGCTGCGAGATT	/138 bp	NM_008361.4
IL-6	TGATGGATGCTACCAAACTGGA<br>TGTGACTCCAGCTTATCTCTTGG	/197 bp	NM_001314054.1
IL-10	GGTTGCCAAGCCTTATCGGA<br>TCAGCTTCTCACCCAGGGAA	/115 bp	NM_010548.2
TNF-α	AGCCGATGGGTTGTACCTTG<br>AGTACTTGGGCAGATTGACCTC	/269 bp	NM_00127860. 1
β-actin	CATCCGTAAAGACCTCTATGCCAAC<br>ATGGAGCCACCGATCCACA	/117 bp	NM_007393.5
