# Supplementary material for: Betulinic Acid Ameliorates T-2 Toxin-Induced Neuroinflammation by Suppressing Oxidative Stress via Regulating Nrf2/NLRP3 Axis
Source: Vet Sci. 2026 May 24;13(6):509. doi: 10.3390/vetsci13060509 (PMC13308106; doi:10.3390/vetsci13060509)
Supplement: Supplementary file 1 [file vetsci-13-00509-s001.zip › vetsci-4296838-original-images.pdf]

β-actin

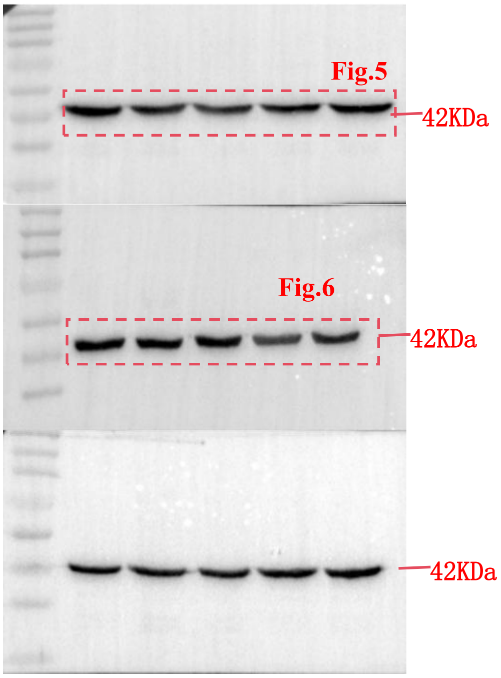

T-2 toxin (mg/kg bw) - 1 1 1 1

BA (mg/kg bw) - - 0.25 0.5 1

Occludin

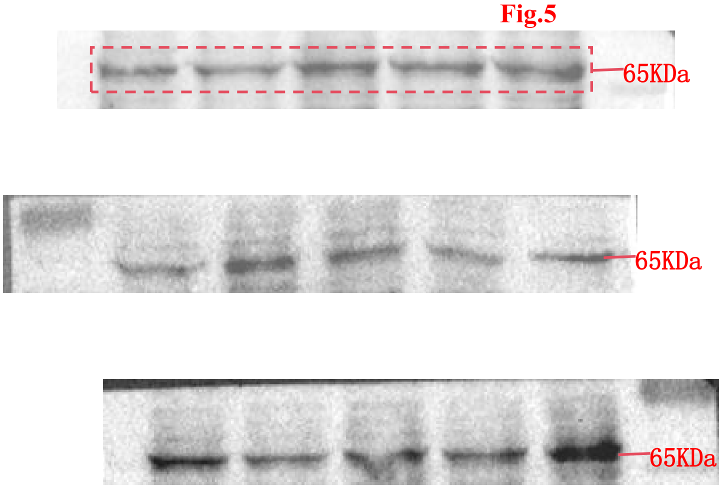

T-2 toxin (mg/kg bw) - 1 1 1 1

BA (mg/kg bw) - - 0.25 0.5 1

Claudin-1

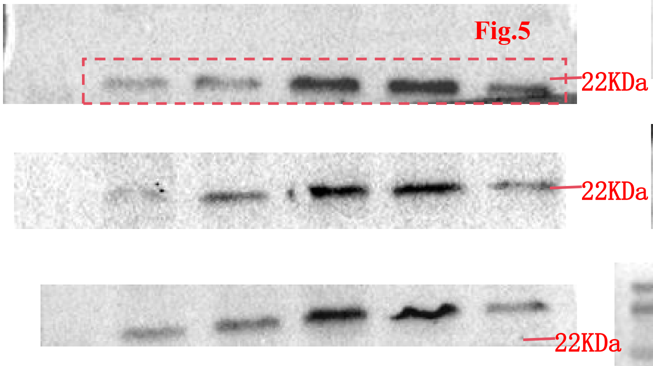

T-2 toxin (mg/kg bw) - 1 1 1 1

BA (mg/kg bw) - - 0.25 0.5 1

NLRP3

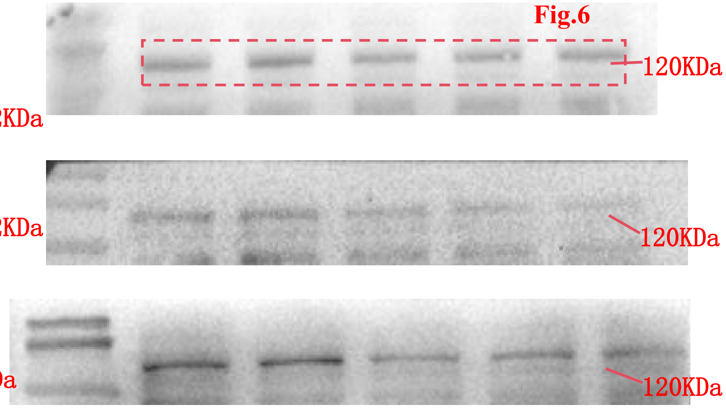

T-2 toxin (mg/kg bw) - 1 1 1 1

BA (mg/kg bw) - - 0.25 0.5 1

ASC

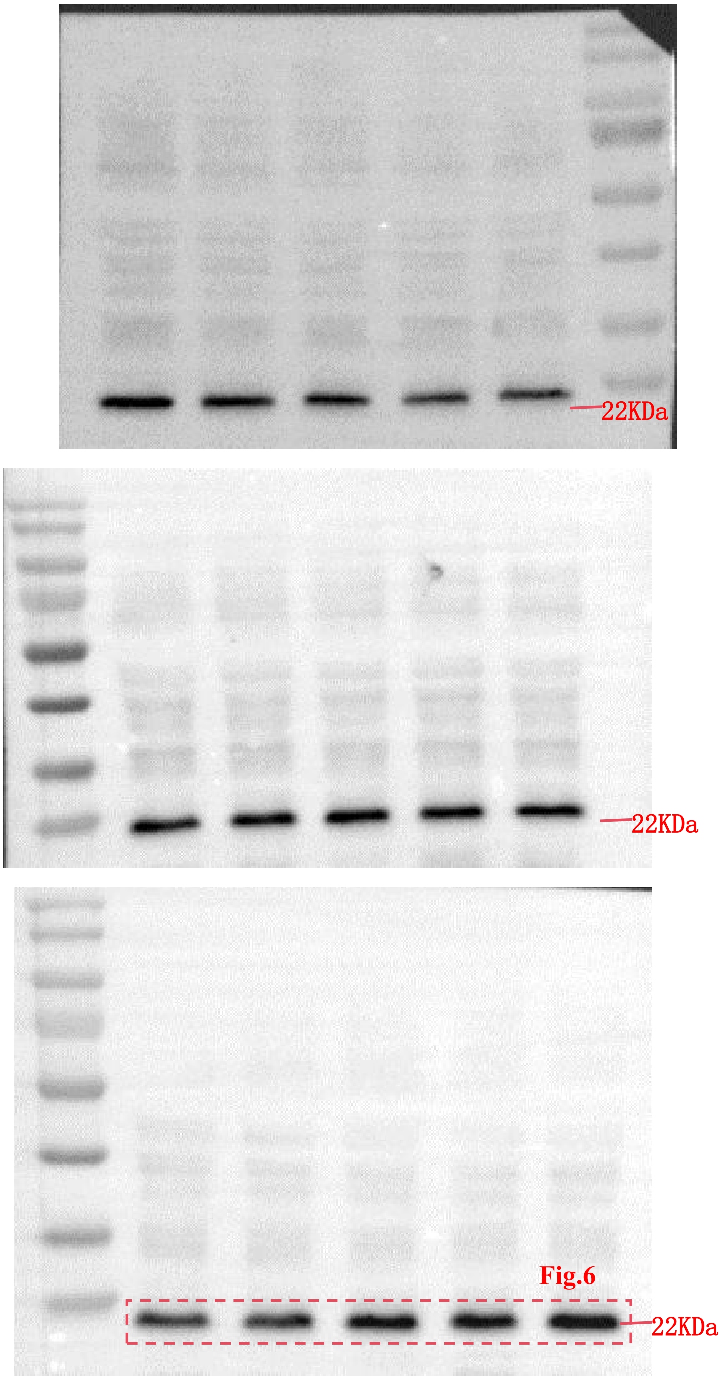

T-2 toxin（mg/kg bw)	-	1	1	1	1
BA（mg/kg bw)	-	-	0.25	0.5	1

Caspase-1

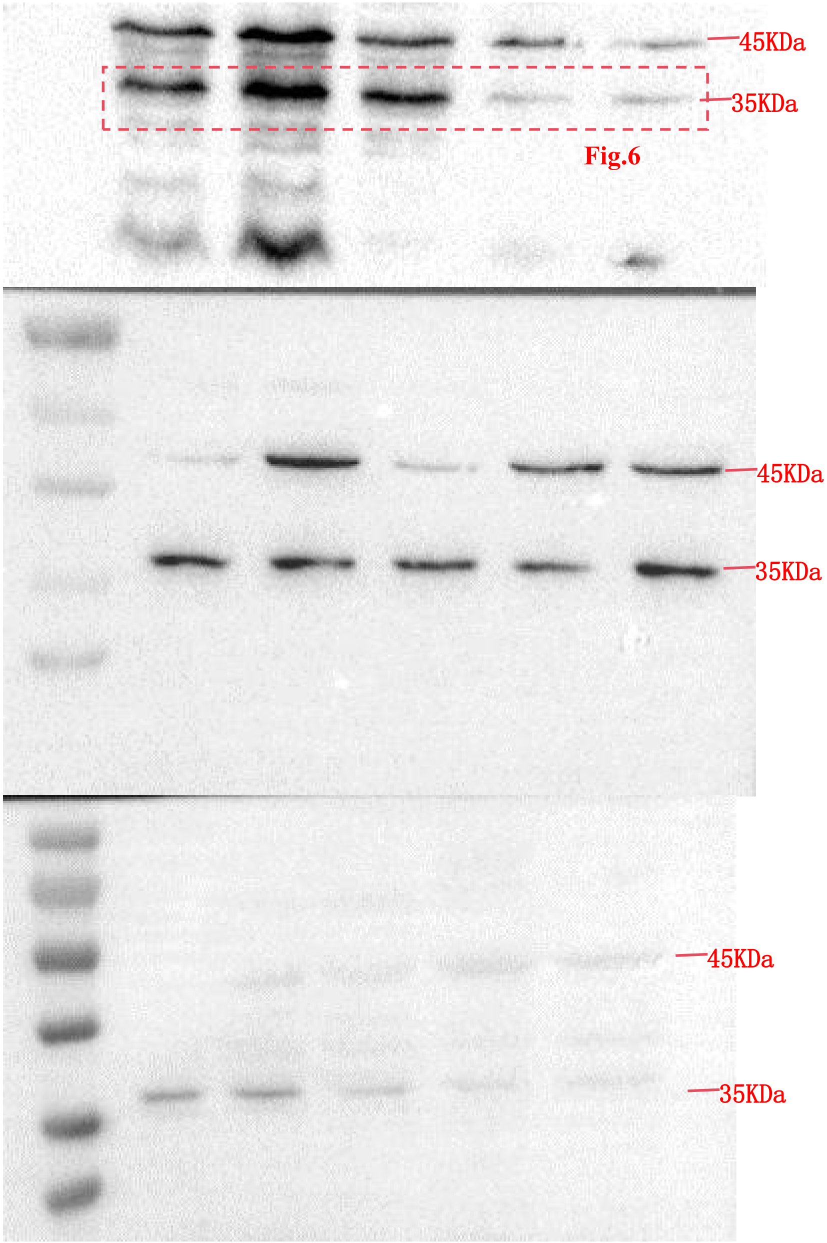

T-2 toxin（mg/kg bw)	-	1	1	1	1
BA（mg/kg bw)	-	-	0．2 5	0．5	1

IL-1β

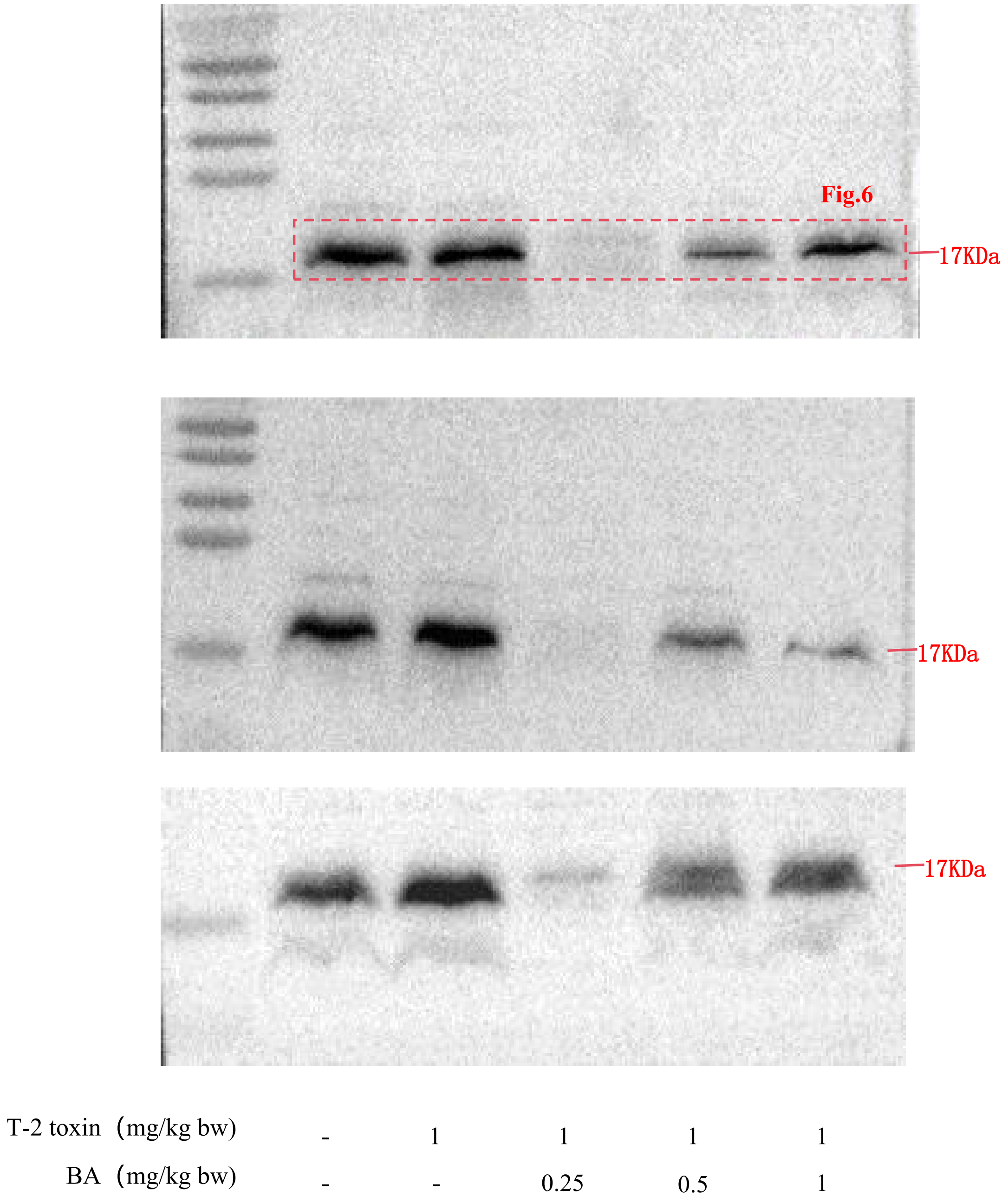
Fig.6
17KDa
17KDa
17KDa
T-2 toxin (mg/kg bw) - 1 1 1 1
BA (mg/kg bw) - - 0.25 0.5 1

IL-18

Fig.6

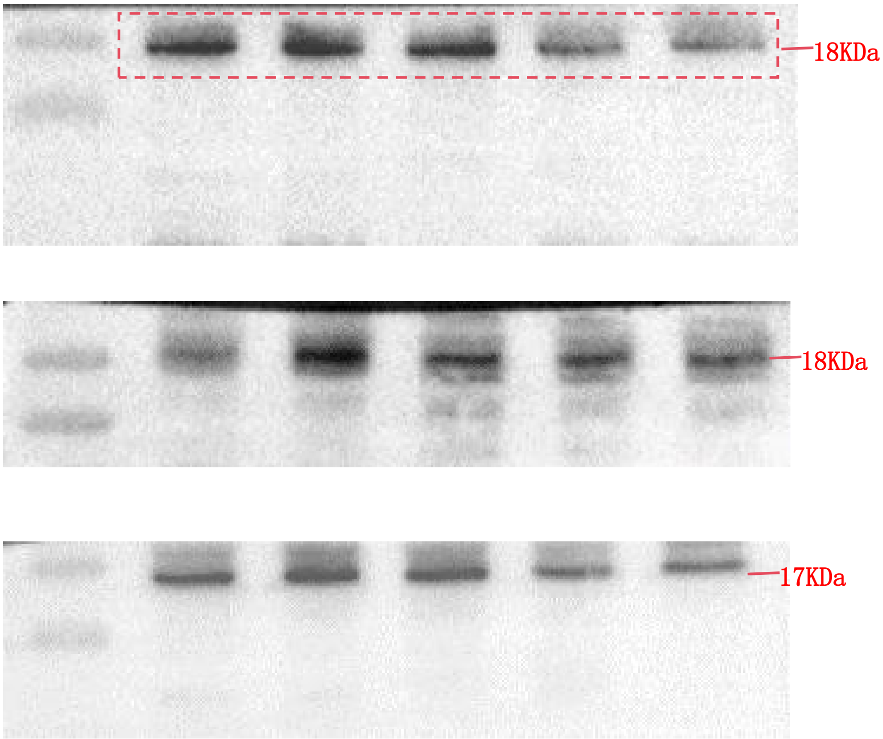

T-2 toxin（mg/kg bw)	-	1	1	1	1
BA（mg/kg bw)	-	-	0.25	0.5	1

Nrf2

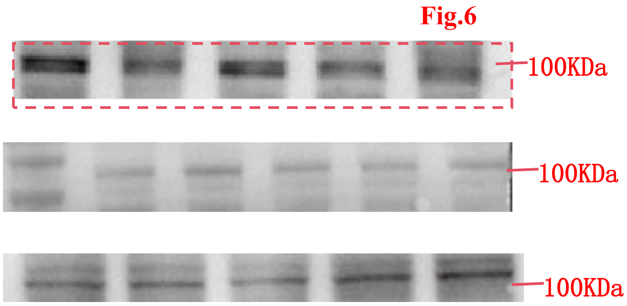

T-2 toxin (mg/kg bw)	-	1	1	1	1
BA (mg/kg bw)	-	-	0.25	0.5	1

Keap1

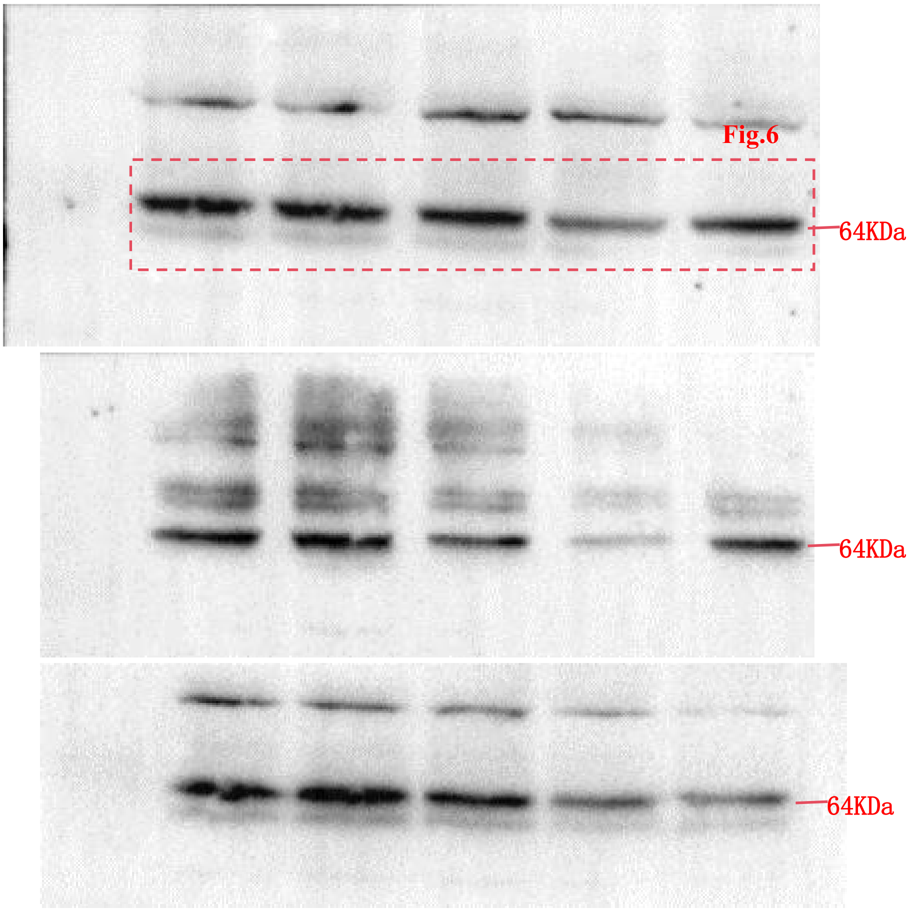

T-2 toxin (mg/kg bw)	-	1	1	1	1
BA (mg/kg bw)	-	-	0.25	0.5	1

HO-1

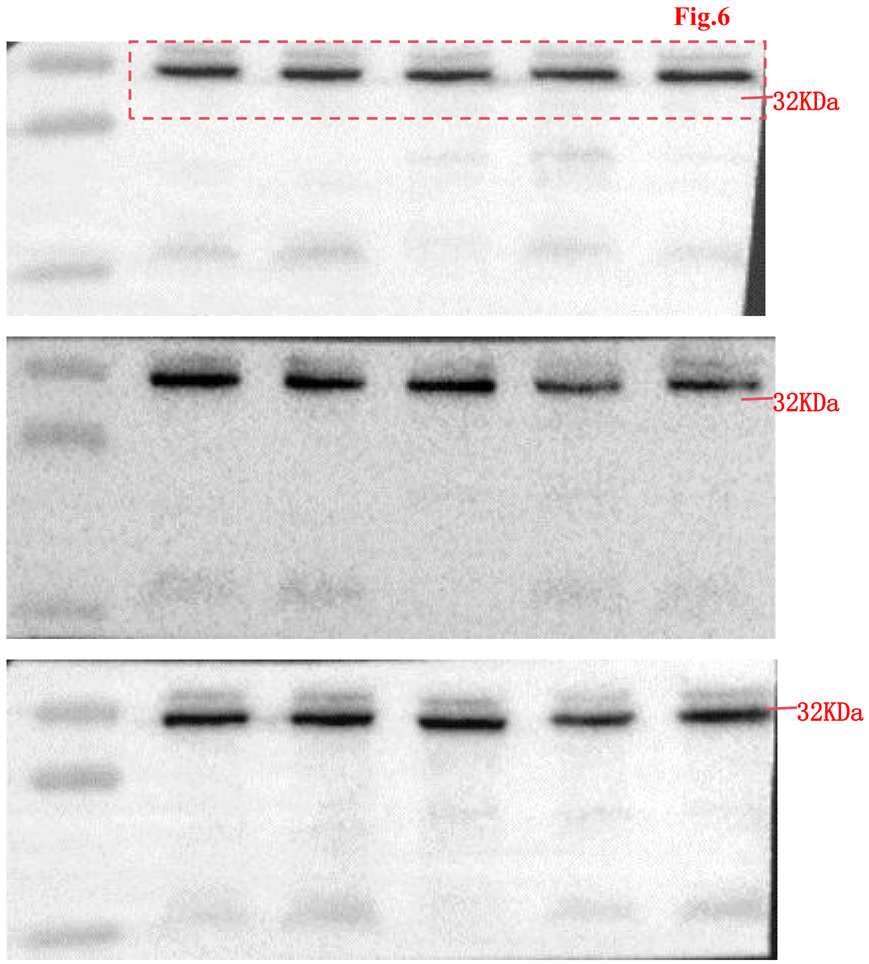

T-2 toxin（mg/kg bw)	-	1	1	1	1
BA（mg/kg bw)	-	-	0.25	0.5	1
